# Tight Regulation of Major Histocompatibility Complex I for the Spatial and Temporal Expression in the Hippocampal Neurons

**DOI:** 10.3389/fncel.2021.739136

**Published:** 2021-10-01

**Authors:** Yuqing Shen, Jianqiong Zhang

**Affiliations:** ^1^Department of Microbiology and Immunology, Medical School, Southeast University, Nanjing, China; ^2^Jiangsu Provincial Key Laboratory of Critical Care Medicine, Department of Critical Care Medicine, School of Medicine, Zhongda Hospital, Southeast University, Nanjing, China; ^3^Key Laboratory of Developmental Genes and Human Disease, Ministry of Education, Southeast University, Nanjing, China; ^4^Jiangsu Key Laboratory of Molecular and Functional Imaging, Medical School, Zhongda Hospital, Southeast University, Nanjing, China

**Keywords:** hippocampus, neuron, gene expression regulation, MHC class I, mice

## Abstract

The expression and function of immune molecules, such as major histocompatibility complex (MHC), within the developing and adult brain have been discovered over the past few years. Studies utilizing classical class I MHC knockout animals suggest that these molecules, in fact, play essential roles in the establishment, function, and modification of synapses in the CNS. Altered neuronal expression of class I MHC, as has been reported in pathological conditions, leads to aberrations in neuronal development and repair. In the hippocampus, cellular and molecular mechanisms that regulate synaptic plasticity have heretofore been extensively studied. It is for this reason that multiple studies directed at better understanding the expression, regulation, and function of class I MHC within the hippocampus have been undertaken. Since several previous reviews have addressed the roles of class I MHC in the formation and function of hippocampal connections, the present review will focus on describing the spatial and temporal expression of class I MHC in developing, healthy adult, and aging hippocampus. Herein, we also review current literatures exploring mechanisms that regulate class I MHC expression in murine hippocampus. With this review, we aim to facilitate a deeper mechanistic understanding into the complex tight regulation of MHC I expression in hippocampus, which are needed as we explore the potential for targeting MHC I for therapeutic intervention in normal aging and in neurodegenerative diseases in the future.

## Introduction

Major histocompatibility complex (MHC) is considered to play central roles in the immune system for presenting peptides and activating lymphocytes. The MHC complex can be divided into three regions: class I, II, and III, and class I MHC genes can be subdivided into classical and non-classical groups (Milner and Campbell, 2001). Over the past decades, the expression of classical class I MHC genes (referred to as MHC I in the following text) within the healthy central nervous system (CNS) has become increasingly recognized and studied. HLA (human leucocyte antigen) is the MHC antigen specific to humans, and HLA-A, HLA-B and HLA-C are the three types of classical human MHC class I proteins. Mouse MHC antigen is called H-2 antigen, and there are three classical mouse class I proteins: H2-K, H2-D, and H2-L. C57BL/6 mice only express two of them: H2-K^b^ and H2-D^b^ (MHC haplotype b) (Tetruashvily et al., 2016). As of today, the roles of classical MHC I proteins in the establishment, function, and modification of synapses have been extensively reviewed (Boulanger and Shatz, 2004; Boulanger, 2009; Shatz, 2009; Fourgeaud and Boulanger, 2010; Elmer and McAllister, 2012; McAllister, 2014). A well-characterized model system for studying neural connection in the CNS is the hippocampus, which is known for its function in regulating learning, memory, and emotion (Martin et al., 2000). Abnormal expression of classical MHC I in the hippocampus resulted in altered synapse number and morphology with irregular synaptic transmission in animal models (Huh et al., 2000; Goddard et al., 2007; Fourgeaud et al., 2010; Wu et al., 2011; Dixon-Salazar et al., 2014; Lee et al., 2014). Since inappropriate classical MHC I expression in hippocampal neurons is deleterious, the expression of MHC I molecules should be maintained under tight regulation. In this review, we focus on describing the spatial and temporal expression of MHC I, especially classical MHC I genes, in the hippocampus of different species, and we also introduce several reports investigating the molecular mechanisms used by hippocampal neurons to regulate MHC I expression.

## Spatial and Temporal Expression of MHC I Molecules in Hippocampus, From Rodent to Primate

### MHC I mRNA and Protein Expression in the Developing and Adult Hippocampus

MHC I expression was generally considered to be limited to non-neuronal cells in healthy uninjured brain (Neumann et al., 1995). In 1998, MHC I mRNA was discovered when researchers screened for genes that were regulated by neural activity in the developing retino-geniculate pathway of cats. These authors also detected MHC I mRNA in hippocampal pyramidal neurons by *in situ* hybridization. During feline development, the expression of MHC I mRNA in hippocampus was found to begin at postnatal day 10 (P10) and was maintained at a relatively high level until P91. The expression of MHC I mRNA and proteins in the dentate gyrus (DG) and cornu ammonis (CA) of normal P22 rat hippocampus was also confirmed by the same group (Corriveau et al., 1998). This was the first report of MHC I molecules that are present normally in subsets of neurons and are required for synapse formation in the LGN.

Since MHC I knockout models were only available in mouse, MHC I expression in the murine hippocampus has been extensively studied. MHC I mRNA in hippocampus was detected in granule and pyramidal cells where P40 mice showed much weaker expression than P6 mice (Huh et al., 2000). Different MHC I alleles showed distinct levels of expression, with H2-D^b^ showing stronger expression in pyramidal cell layers of hippocampus than that of H2-K^b^ (Huh et al., 2000). By checking with *in situ* hybridization using specific probes, our group also observed that H2-D^b^ mRNA was expressed postnatally (P4) in CA, DG, and also in the mossy fiber of the hilus in mouse hippocampus. H2-K^b^ signals ran parallel with H2-D^b^, and their expression levels were almost identical. Evidence of expression continued at P8 and P15, while at P30 and in adult mice, the signal decreased and became limited to DG and CA1 regions of hippocampus (Liu et al., 2013). Since the expression level of MHC I alleles we reported during hippocampal development differs from other reports, we later measured H2-K^b^ and H2-D^b^ expression using qRT-PCR, a more quantitative method. The result demonstrated that MHC I mRNA increased steadily from P0 to P60 in mouse hippocampal tissues with higher expression of H2-K^b^ than H2-D^b^, suggesting an uneven expression of different MHC I alleles as other reports (Li et al., 2020). Meanwhile, we also detected MHC I proteins by immunofluorescence in hippocampal neurons, but not in GFAP-positive astrocytes (Lv et al., 2015; Li et al., 2020). While the mRNA levels of MHC I increased steadily, the change of MHC I proteins only mirrored MHC I mRNAs levels from P0 to P15. After that, expression of MHC I proteins declined sharply at P30 and P60, suggesting a posttranscriptional regulation (Lv et al., 2015; Patel et al., 2020). Detailed cell-type-specific difference in mouse MHC I gene expression within the hippocampus were also recently identified. Multiple MHC I genes were found to be transcribed in hippocampal tissues collected from P13 to P16. A single-cell mRNA-based approach was used to examine neurons collected from hippocampal tissue. These studies revealed that H2-K^b^ and H2-D^b^ mRNA were largely present in CA1 pyramidal neurons. Expression profiles of MHC I mRNA in CA1 were significantly different from those of CA3 and DG, which helped to clarify that certain phenotypes, such as increased synapse number, were only observed in restricted regions of the hippocampus of the systemic MHC I knockout mice (Tetruashvily et al., 2016). In general, MHC I gene expression is confirmed in the developing murine hippocampus. However, individual hippocampal neurons can express more than one MHC I gene and their gene expression profiles differ significantly from each other.

The organization, structure, and function of many genes can be quite different from rodents to primates. Therefore, the expression of MHC I proteins in higher vertebrates was investigated. We collected fixed human brain tissues from aborted human fetus, infants, and adults who died without major pathological findings in the CNS (Zhang et al., 2013). Since these specimens were already fixed with paraformaldehyde, immunohistochemistry was used to detect MHC I protein expression by two fully validated allele-specific anti-MHC antibodies: HC-10 and HLA-A2, which recognized HLA-B/C and HLA-A proteins, respectively, in fixed human tumor tissue (Patel et al., 2020). In human hippocampus, MHC I expression was very low at 20 gestational weeks (GW), slowly increased at 27–33 GW, and was maintained to postnatal 55 days. We cannot detect any MHC I protein expression in neurons of the adult brain. Just as reported in the rodents, MHC I proteins were mainly expressed in neurons but not in astrocytes, with most MHC I-expressing neurons being glutamatergic (Zhang et al., 2013). Because human fetal brain tissue is often unavailable, there is only one study about MHC I expressing in the development of human brain. Further studies on the human brain are highly required before we can draw any conclusions. In common marmosets, a non-human primate, expression of classical MHC I proteins in hippocampus was also detected by immunostaining with HC-10 and HLA-A2 antibodies (Rolleke et al., 2006; Ribic et al., 2010). MHC I expression was within mossy fibers and co-localized with vGlut1 immuno-positive signals, suggesting a role of MHC I proteins in excitatory transmission at mossy fiber-CA3 synapses. The expression of MHC I proteins was found to be identical from 1 day postnatal to 6 years old in common marmoset monkeys. In addition, the expression of MHC I mRNAs was examined by *in situ* hybridization. However, the authors concluded that the signal detected belongs to non-classical MHC I molecules.

### MHC I mRNA and Protein Expression in the Aging Hippocampus

An age-related increase in MHC I mRNA was observed in the synaptosomal parts of hippocampus neuron in aged rats (26–28 months old) compared with the adult rats (12 months old). The expression level of MHC I protein was also increased in aged rats as compared to adult rats (VanGuilder Starkey et al., 2012). To date, most researchers believe that the increased expression of MHC I mRNA and proteins in aged animals is important in regulating synaptic plasticity and cognitive function. Complete absence of MHC I (constitutive knockout of H-2K/D, β2M, or TAP) resulted in decreased dendritic complexity, altered dendritic spine type and increased GluN2B NMDA receptor in CA1 pyramidal neurons in aged mice (Lazarczyk et al., 2016). β2M, light chain of MHC I complex, is elevated in the blood of aging humans and mice and promotes age-related cognitive dysfunction and impairs neurogenesis, raising the possible therapies targeting β2M for treatment of age-related cognitive dysfunction (Smith et al., 2015). However, whether MHC I proteins are increased in neurons of aging human and mouse hippocampus and how MHC I is involved in age-related cognitive loss remain unknown.

## Regulation of MHC I Expression in Mouse Hippocampal Neurons

Since MHC I has been reported to play an essential role in the establishment and modification of neuronal connections in the hippocampus, it must be under tight regulation. MHC I was discovered among a set of genes whose expression was decreased when endogenous action potential activity was blocked by intracranial infusion of TTX; thus, its expression was determined to be regulated by neural activity (Corriveau et al., 1998). Several reports have additionally indicated that MHC I expression is significantly regulated by seizure-induced overexcitation in the hippocampus (Corriveau et al., 1998; Lee et al., 2021). However, the signaling pathways that relay neuronal activity to MHC I expression have remained poorly defined.

### Transcriptional Regulation of MHC I Expression by Neural Activity

The regulation of MHC I expression has been extensively studied in immune cells and in other cell types. It has been determined that tissue-specific expression of MHC I is regulated by distal and proximal upstream regulatory elements, which are located at −800 to −700 bp and −220 to −95 bp regions of the promoters, respectively (Howcroft et al., 2005). In cell types with low endogenous MHC I expression, a silencer element in the promoter distal regulatory elements has been found showing increased activity (Weissman and Singer, 1991). For example, in one such cell line, human neuroblastoma CHP-126, the absence expression of MHC I was a direct result of repressor activity in upstream silencer elements (Murphy et al., 1996).

Although neurons were considered to be deficient in MHC I expression before 1998, they have been found to react to cytokine treatment, such as IFN-γ and TNF-β, by increasing MHC I transcription, suggesting their ability to quickly induce MHC I expression under pathogenic conditions and to activate the immune system (Neumann et al., 1997). Besides differences in silencer activity within the promoter distal regulatory elements, basal and activated transcription of MHC I genes is dependent on different transcriptional factors binding to the core promoter domain and is initiated at different sites within the MHC I promoter (Lee et al., 2010). It is unknown whether neural activity could induce MHC I transcription *via* a similar mechanism used by cytokines. In neurons, changes in activity always result in a remodeling of the strength of synapses (also called activity dependent plasticity), which involves a series of gene transcriptional changes (Gainey et al., 2015; Joseph and Turrigiano, 2017). The transcription factor CREB has been reported to be necessary for activity-dependent plasticity in hippocampal neurons (Josselyn and Nguyen, 2005) and has also been reported to be one of the key regulators of MHC I expression in immune cells (Meissner et al., 2012). Thus, CREB may be a central player in neural-activity-induced MHC I transcription. We reported on the induction of MHC I expression in cultured hippocampus neurons by enhanced neural activity with KA treatment. We also have shown that transient intracellular Calcium [Ca^2+^] flux induced by KA activated protein kinase A and C, which served as upstream regulators to increase the amount of NF-κB, IRF-1, and CREB translocation into the nucleus and finally upregulated MHC I transcription. Thus, membrane activity can be connected to neuronal MHC I expression through the modulation of intracellular Ca^2+^ concentration and Ca^2+^ regulated signal transduction cascades that share the same transcription factors as those induced by cytokines (Lv et al., 2015). In short, transcription of MHC I can be regulated by tissue-specific (basal) and cytokine/membrane activity (activated) mechanisms in hippocampal neurons. However, our study was performed using cultured neurons with KA treatment that can induce action potentials. Whether changes in physiological level of neural activity in hippocampal neurons *in vivo* during specific development stages activate the same pathways to regulate MHC I transcription requires further investigation.

### Transcriptional Regulation of MHC I Expression by NLRC5, a New Transcriptional Regulator in Hippocampal Neurons

**FIGURE 1:**
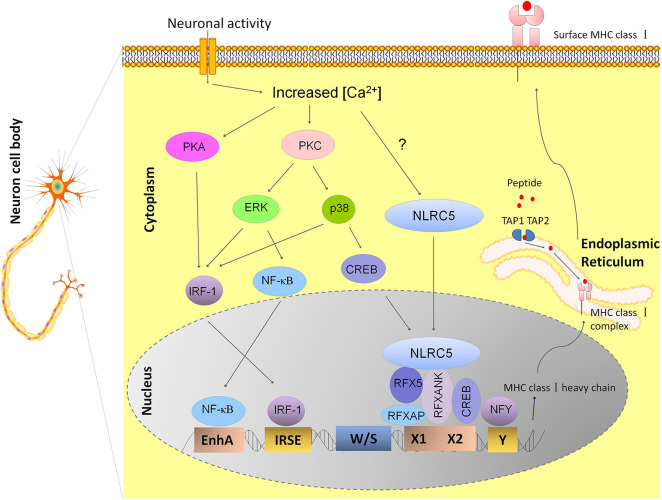
Model depicting the transcriptional regulation of MHC I expression in hippocampal neurons. The transcriptional regulation of MHC I genes is dependent on different transcriptional factors binding to the conserved DNA sequence motifs within the MHC I promoter. Neuronal activity may leads to activation of calcium-dependent Protein Kinase A and C (PKA and PKC), thus results in activation of ERK and P38 of the MAPK pathways and increases the nuclear translocation of transcription factors NF-κB, CREB, and IRF-1. These molecules finally leads to enhanced expression of MHC I by binding to its promoter elements: enhancer A, ISRE, and X box, respectively (Lv et al., 2015). RFX complex also binds to X box besides CREB and the nuclear transcription factor Y (NFY) binds to the Y box. NLRC5 is recruited to the enhanceosome by RFX complex. However, how neuronal activity affects NLRC5 is still not known (Li et al., 2020). Newly synthesized MHC class I heavy chains and beta-2 microglobulin (β2m) proteins form MHC I complex with antigenic peptides in endoplasmic reticulum (ER) and MHC I complex loaded with antigens are transported to the cell surface.

The expression profile of MHC I in neurons behaves in much the same way as classical class II MHC molecules (MHC II) in the peripheral immune system, with cell-type-specific expression and inducible expression by cytokines (Muhlethaler-Mottet et al., 1997). Class II major histocompatibility complex transactivator (CIITA) is the key factor that determines the expression profile of MHC II (Accolla et al., 2019). However, along with other groups, we have also reported that CIITA is not found expressed in normal neurons, so it does not determine the spatial and temporal expression of MHC I in neurons (Castillo et al., 2008; Li et al., 2020). NLRC5, a recently defined transactivator of MHC I genes, attracted our attention for its functional similarity to CIITA that coordinates other transcription factors to form an enhanceosome on the MHC I promoter (Meissner et al., 2010). Abolishing NLRC5 expression resulted in reduced MHC I expression in several immune cells, although to different extents (Biswas et al., 2012; Staehli et al., 2012). Just as has been shown in the immune system, we identified dramatically decreased MHC I mRNA expression in the hippocampus from P0 to P60 in NLRC5 knockout mice, suggesting that NLRC5 is a key regulator of constitutive neural MHC I expression. In the murine neuroblastoma cell line, Neuro2a, NLRC5-induced MHC I activation requires the recruitment of NLRC5 to other transcriptional factors that bind to W/S-X-Y promoter domains (Li et al., 2020). Besides the activation of MHC I transcription, other molecules involved in surface MHC I expression such as β2-microgloubulin, TAP, and LMP are also regulated by NLRC5 in Nero2a cells, implying the potential value of NLRC5 as a target for manipulating neuron MHC I expression (Li et al., 2020). In summary, the working model of transcriptional regulation of MHC I expression in hippocampal neuron is shown in Figure 1.

### Posttranscriptional Regulation of MHC I Expression in Neurons

Although the expression of MHC I molecules is in large part transcriptionally regulated, the dissociated expression of MHC I mRNA and proteins from P15 to P60 in mouse hippocampus indicates the existence of posttranscriptional regulatory mechanisms. In the immune system, MHC I expression is regulated by microRNAs, RNA binding proteins, and ubiquitin E3 ligase, which affects mRNA stability or indirectly changes the translation efficiency (Kulkarni et al., 2011, 2013; Reches et al., 2016). The ubiquitination system also targeted MHC I proteins to regulate protein transport or proteasomal degradation in the cytosol (Cano et al., 2012). The expression of miR-34a increases rapidly at P30 and P60 in mouse hippocampal tissue, making it an ideal candidate for posttranscriptional regulation of MHC I expression in hippocampal neurons. As we expected, miR-34a showed the ability to regulate MHC I expression in cultured hippocampus neurons. Since MHC I mRNA and protein expression were both decreased upon enhanced expression of miR-34a, we cannot tell whether miR-34a exerts its function through reducing the stability of MHC I mRNA or inhibiting the protein translation (Hu et al., 2020). However, additional *in vivo* data exploring the regulation of MHC I by miR-34a are still needed.

## Discussion

In summary, the expression of MHC I mRNA and protein in postnatal, adult, and aging hippocampal neurons of several species has been detailed by multiple independent studies (Table 1). However, reports of dynamic expression of MHC I molecules in hippocampus throughout the entire life span (prenatal to aged adult) of one species are scarce since most research focuses only on specific developmental stages. Another shortcoming is that the expression of MHC I proteins in mouse neurons remains to be further validated since antibodies that can specifically recognize mouse MHC I are lacking. Most anti-mouse MHC I antibodies available today are only suitable for detecting the fully folded MHC I proteins *in situ* at the cell surface, and they are not useful for detecting denatured MHC I proteins by Western blots or by immunofluorescence (Wu et al., 2011). Although there have been reports describing a new way for surface labeling of MHC I proteins by immunofluorescence in cortical neurons (Glynn et al., 2011), the staining steps still involved the fixation of coverslips, and using these antibodies for immunofluorescence was not widely accepted. OX18 is the common antibody used for studying neuronal MHC I in rodents, and it can recognize native and denatured rat and mouse MHC I molecules. However, it recognizes both classical and non-classical MHC I molecules (Needleman et al., 2010; Wu et al., 2011). Generating and validating new anti-MHC I antibodies that can be used for detecting denatured MHC I proteins in mice is necessary for precisely determining how protein expression corresponds to the abundance of MHC I mRNAs in neurons. Furthermore, high-specificity antibodies that can accurately discriminate between individual members of the MHC I family will be indispensable for identifying which MHC I genes participate in the establishment and refinement of hippocampal circuits. Finally, it remains important to accurately define the expression of MHC I proteins across different regions of the hippocampus since abnormal synapse structure and function have been observed in specific parts of the hippocampus in global MHC I knockout mice and in H2-D^b^ overexpression mice (Wu et al., 2011; Dixon-Salazar et al., 2014). In order to clearly elucidate this, studies favoring regional or single-cell hippocampal neurons should be prioritized over whole hippocampus homogenates.

**TABLE 1:** MHC I mRNA and protein expression in hippocampus.

**Species**	**Sample size**	**Class I MHC expression**	**Allele analyzed**	**Methods**	**References**
Feline	Not available	Begin at p10 (postnatal day 10), maintain high level to p91.	MHC I	ISH	Corriveau et al., 1998
Rats	Not available	In DG and CA1/CA3 pyramidal cell of P22 rat hippocampus	MHC I	ISH, ICC	Corriveau et al., 1998
Aged Rats	24 adult and 134 aged rats	Significant increased with aging when compared aged rats (28 month old) with adult rats (12 month old). MHC I was elevated with aging in synaptosomes, CA1, and DG of hippocampus.	Classical MHC I (RT-1A) (RT-qPCR), MHC I (WB,IF)	Subregion dissection, Synaptosome Preparation, qRT-PCR, WB, IF	VanGuilder Starkey et al., 2012
Mice	Not available	Signals in granule and pyramidal cells of P6 hippocampus, with mRNA level declined at P40. H-2K signal paralleled that of H-2D, but was much lower.	Classical MHC I (H–2D, H–2K) and non-classical MHC I (Qa-1, T22)	ISH	Huh et al., 2000
Mice	E10.5, E12.5, E16.5, P4, P8, P15, P30, and adult mice were investigated (5 mice at each stage)	From P4 to P15, the signal was gradually increased and decreased from then on. Signal was remarkably detected in the DG, CA and mossy cell.	Classical MHC I (H–2D, H–2K)	ISH, IF	Liu et al., 2013
Mice	Not available	Hippocampal neurons expressed multiple MHC I genes, with different profile in CA1, CA3 pyramidal neurons and DG granule cells.	Classical MHC I (H–2D, H–2K) and multiple non-classical MHC I alleles	Subregion dissection, Single-cell qRT-PCR	Tetruashvily et al., 2016
Marmoset Monkeys	6 (1 female and 5 males)	Signals was strong in pyramidal neurons of the hippocampal formation and in granule neurons of DG in adult common marmoset monkeys.	MHC I	ISH, WB, FC, IF, IHC	Rolleke et al., 2006
Marmoset Monkeys	15 (14 males and 1 female)	MHC I signal was solely on the nerve terminals of mossy fibers and the distribution was identical from 1 day to 6 year old.	Classical MHC I	WB, IHC, IF	Ribic et al., 2010
Homo Sapiens	20 human brain tissues: 17 from fetus (2 at 20GW, 1 at 23GW, 2 at 25GW, 3 at 26GW, 2 at 27GW, 1 at 29GW, 2 at 30GW, 2 at 31GW 2 at 33GW), 1 55-day-old infant and 2 adults (age not known)	Signal was very low at 20 gestational week, reached the peak at 30-33GW and disappeared in adult. Expressed by granular cells in CA and DG and mainly located in glutamatergic neurons.	Classical MHC I (HLA-A and HLA-B/C)	IHC, IF	Zhang et al., 2013

*FC, flowcytometry; GW, Gestational weeks; ICC, Immunocytochemistry; IF, Immunofluorescence; IHC, Immunohistochemistry, ISH, In situ hybridization; MHC I, class MHC I molecules; WB, Westenblotting.*

Transactivation of MHC I is normally suppressed in neurons, but it was readily inducible in response to stimuli such as cytokines or neural activity (Neumann et al., 1995). After the critical developmental time frame, reduction of neural activity can decrease MHC I expression, suggesting that this is a very dynamic system. In the development of murine hippocampus, MHC I protein level was dramatically decreased after P15 while MHC I mRNA was kept at high level from P0 to P60 (Li et al., 2020). We confirmed that NLRC5 was responsible for promoting constitutive transcription of MHC I gene, and Ca^2+^-regulated signal transduction cascades were important for relaying membrane activity to upregulated MHC I expression in hippocampal neurons (Lv et al., 2015; Li et al., 2020). We also found that miR34a, which increased dramatically after P15, can decrease MHC I expression, suggesting that it contributed to divergent expression of MHC I mRNA and protein expression after P15 (Hu et al., 2020). Although some mechanisms have been discovered, the information about the pathways that are responsible for temporal neuronal MHC I expression is still very limited. First, most of these results were performed in *in vitro* cultured system. Bear in mind that results may be in opposition when studied using *in vitro* vs. *in vivo* systems. Furthermore, cell-type-specific difference in MHC I gene expression within the hippocampus indicates that regulatory mechanisms may differ between neurons located within distinct regions of the hippocampus. In addition, posttranscriptional regulation could not be excluded since there is disparity between MHC I mRNA and protein expression. Besides the regulation by miRNA, ubiquitination of the MHC I cytoplasmic tail was described to quickly change the surface expression of MHC I proteins in immune system (van den Boomen and Lehner, 2015). This could be a way by which neurons respond to alterations in neural activity and subsequently result in formation of precise neural connections. There remain many unanswered questions regarding the regulation of MHC I in hippocampal neurons through different developmental stages and in aged animals. The answers to these questions remain very important for the field to more clearly understand the diverse roles of MHC I molecules in normal neuronal development and also in age-related cognitive dysfunction or neurodegenerative diseases.

## Author Contributions

YS: contributions to the design and drafting of the manuscript and agrees to be accountable for all aspects of the work in ensuring that questions related to the accuracy or integrity of any part of the work are appropriately investigated and resolved. JZ: contributions to the revise the manuscript critically for important intellectual content. Both authors contributed to the article and approved the submitted version.

## Conflict of Interest

The authors declare that the research was conducted in the absence of any commercial or financial relationships that could be construed as a potential conflict of interest.

## Publisher’s Note

All claims expressed in this article are solely those of the authors and do not necessarily represent those of their affiliated organizations, or those of the publisher, the editors and the reviewers. Any product that may be evaluated in this article, or claim that may be made by its manufacturer, is not guaranteed or endorsed by the publisher.

## Abbreviations

CA: cornu ammonis
CNS: central nervous system
DG: dentate gyrus
CREB: cAMP response element binding protein
CIITA: class II major histocompatibility complex transactivator
GW: gestation weeks
IRF-1: interferon regulatory factor 1
KA: kainic acid
LMP: low-molecular-weight peptide
LGN: lateral geniculate nucleus
MHC: major histocompatibility complex
NF-κB: nuclear factor kappa B
NLRC5: NLR family CARD domain containing 5
RFX: regulatory factor X
RFXANK: regulatory factor X-associated ankyrin-containing protein
TAP: transporter associated with antigen processing
TTX: Tetrodotoxin.
